# Fine‐Needle Aspiration of Salivary Gland Tumors With Low‐Grade Oncocytic Features: Focusing on Oncocytoma Versus Oncocytic Mucoepidermoid Carcinoma

**DOI:** 10.1002/dc.70202

**Published:** 2026-08-25

**Authors:** Lois Araneta, Ying‐Ju Kuo, Jen‐Fan Hang

**Affiliations:** ^1^ Rizal Medical Center Pasig City Metro Manila Philippines; ^2^ Chinese General Hospital College of Medicine Manila Philippines; ^3^ Department of Pathology and Laboratory Medicine Taipei Veterans General Hospital Taipei Taiwan; ^4^ Department of Pathology, School of Medicine National Yang Ming Chiao Tung University Taipei Taiwan

## Abstract

**Background:**

Salivary gland fine‐needle aspirates (FNAs) with low‐grade oncocytic features are diagnostically challenging due to the substantial morphologic overlap between benign oncocytic tumors and their malignant mimickers. This study evaluates the diagnostic discriminators, performance, and risk of malignancy of these lesions using the Milan System for Reporting Salivary Gland Cytopathology (MSRSGC).

**Methods:**

We retrospectively reviewed salivary gland FNA cases (2021–2025) categorized as Milan IVa: Benign neoplasm with oncocytic features, including Warthin tumor and oncocytoma/nodular oncocytic hyperplasia (NOH), and Milan IVb: Salivary gland neoplasm of uncertain malignant potential (SUMP) with oncocytic features, with surgical follow‐up. Cytomorphological analysis was performed on liquid‐based cytology (LBC), direct smears, and cell blocks. Features including architectural patterns, cytological details, and background were correlated with histology.

**Results:**

Forty‐nine cases were included. All 43 cases diagnosed as Milan IVa were confirmed as Warthin tumor or oncocytoma/nodular oncocytic hyperplasia. Warthin tumor shows cohesive, flat clusters of oncocytes along with a cystic debris‐like background, while oncocytoma/NOH shows compact globoid clusters of oncocytes with a “bunch of grapes” pattern and clean background. In the six Milan IVb cases, follow‐up histology included oncocytic mucoepidermoid carcinoma (OMEC, *n* = 2), oncocytoma/nodular oncocytic hyperplasia (*n* = 2), oncocytic lipoadenoma (*n* = 1), and spindle cell lipoma (*n* = 1). The OMEC cases form discohesive clusters and single cells. Cell block preparations revealed focal glandular structures and rare intracytoplasmic mucin.

**Conclusion:**

The MSRSGC effectively risk‐stratifies low‐grade oncocytic salivary gland lesions. While cohesive, organoid architecture supports a benign diagnosis, the presence of isolated cells and discohesive clusters is better classified as SUMP. Cell block preparation is useful for identifying mucin and glandular differentiation.

## 1 Introduction

Salivary gland neoplasms present a diagnostic challenge due to their morphological and genetic diversity. Despite their complexity, salivary gland neoplasms comprise only 3 to 10% of head and neck neoplasms [1, 2]. Oncocytic neoplasms exemplify this diagnostic difficulty, often obscuring the distinction between benign and malignant entities, as both can present with a low‐grade morphology in routine cytological preparations. Consequently, traditional cytologic features of malignancy, such as high‐grade nuclear atypia and tumor necrosis, are insufficient for establishing a definitive distinction. Furthermore, the relative rarity of these lesions often results in limited diagnostic experience, leading to uncertainties and potential pitfalls that may ultimately impact clinical management [3]. This underscores the importance of refining cytological criteria specifically for lesions with low‐grade oncocytic features.

Fine‐needle aspiration (FNA) remains the primary diagnostic modality for salivary gland lesions, offering high sensitivity (86%–100%) and specificity (90%–100%) in differentiating non‐neoplastic from neoplastic processes [4, 5]. However, diagnostic accuracy ranges from 48% to 94% when attempting to subclassify specific lesions [6, 7]. The implementation of the Milan System for Reporting Salivary Gland Cytopathology (MSRSGC) has further provided a standardized framework for risk stratification [8]. Despite this, oncocytic tumors continue to represent a significant source of diagnostic challenge, often falling into the “Salivary Gland Neoplasm of Uncertain Malignant Potential (SUMP)” category [9, 10, 11]. Key differential diagnoses include Warthin tumor, oncocytoma, oncocytic mucoepidermoid carcinoma (OMEC), and other salivary lesions with oncocytic features [8].

To address these diagnostic pitfalls, this study evaluates the cyto‐histologic correlation of low‐grade oncocytic lesions within a single‐institution series, focusing on the identification of specific diagnostic determinants that distinguish Milan Category IVa (Benign neoplasm) from IVb (SUMP) cases. Furthermore, we examine the utility of cell blocks in bridging the gap between cytologic suspicion and definitive histologic diagnosis.

## 2 Materials and Methods

### 2.1 Case Selection

We retrospectively reviewed salivary gland cytopathology cases from 2021 to 2025, identifying those diagnosed as Milan IVa: Benign neoplasm with oncocytic features, including Warthin tumors and oncocytoma/nodular oncocytic hyperplasia (NOH), and IVb: SUMP with oncocytic features, with follow‐up surgical pathology via an electronic database search. Cytologic and histologic diagnoses, including results from ancillary testing (immunohistochemical stains and molecular studies), were recorded. Demographic data, including age, sex, and history of malignancy, were also collected, alongside relevant imaging modalities and findings.

### 2.2 Cyto‐Histologic Correlation

The evaluation consisted of a comprehensive review of all cytological specimens validated by two board‐certified cytopathologists. The evaluation was focused on three primary features: (1) architectural patterns; (2) cytologic details, specifically nuclear and cytoplasmic morphology; and (3) secondary/background features, including necrosis and associated cell populations. To establish diagnostic discriminators, the cytomorphological profiles of benign cases were compared with those of histologically confirmed malignant tumors with oncocytic features.

## 3 Results

### 3.1 Patient Characteristics

During the study period, a total of 822 fine‐needle aspirations (FNAs) were reviewed. One hundred sixty‐nine cases (20.6%) were selected for analysis: 141 were classified as Milan IVa: Benign neoplasm, Wartin tumor (17.1%), 14 (1.7%) as Milan IVa: Benign neoplasm, oncocytoma/NOH, and 14 (1.7%) as Milan IVb: SUMP, with oncocytic features. After excluding cases without surgical follow‐up, the final series comprised 49 cases (43 from Category IVa and six from Category IVb). All specimens were processed using BD SurePath liquid‐based cytology (LBC) and two direct smears stained with Papanicolaou and Liu's stain. Cell blocks were available for 8 out of the 49 cases (16%).

The patient characteristics are summarized in Table 1. The age ranged from 41 to 82 years, with a male‐to‐female ratio of 41:8. The parotid gland was the most common site (*n* = 47), followed by the submandibular gland (*n* = 2). All cases were assessed by ultrasonography, showing hypoechoic nodules measuring 0.8 to 4.7 cm. The majority have well‐defined margins on ultrasound except for 2 cases with ill‐defined margins. Five cases were accompanied by CT scans, showing solitary to multinodular, well‐defined enhancing lesions. A slight discrepancy of tumor size and multinodularity between sonography and CT was noted.

**TABLE 1:** Clinical information and patient characteristics.

Age	41 to 82 (median: 62)
Sex	Male (*n* = 41)
	Female (*n* = 8)
Site of lesion	Parotid (*n* = 47)
	Submandibular (*n* = 2)
Preoperative FNA Diagnoses	Milan IVa, Benign, Warthin tumor (*n* = 39)
	Milan IVa, Benign, oncocytoma/NOH (*n* = 4)
	Milan IVb, SUMP, with oncocytic features (*n* = 6)
Histologic diagnoses	Warthin tumor (*n* = 39)
	Oncocytoma (*n* = 3)
	NOH (*n* = 3)
	OMEC (*n* = 2)
	Spindle cell lipoma (*n* = 1)
	Oncocytic lipoadenoma (*n* = 1)

Abbreviations: NOH, nodular oncocytic hyperplasia; OMEC, oncocytic mucoepidermoid carcinoma; SUMP, salivary gland neoplasm of uncertain malignant potential.

### 3.2 Histologic Diagnosis and Risk of Malignancy (ROM)

In the Milan IVa group, all 39 cases were confirmed to be Watrthin tumor and all four cases were confirmed to be oncocytoma or NOH in the follow‐up excision. This yielded a concordance rate of 100% and a ROM of 0%. Within the Milan IVb group (*n* = 6), four cases were benign upon excision, which included two oncocytomas, one spindle cell lipoma, and one oncocytic lipoadenoma. The remaining two cases were histologically identified as OMEC and molecularly confirmed by *MAML2* break‐apart fluorescence in situ hybridization (FISH), resulting in an overall ROM of 33.3%.

### 3.3 Cyto‐Histologic Correlation

The cytomorphologic features for each case are summarized in Table 2. In Milan Category IVa, Warthin tumor cases typically demonstrate cohesive, flat clusters of oncocytic cells, accompanied by cystic degenerative debris and a lymphocyte‐rich background (Figure 1A,B). The nuclei are round and uniform, with occasional prominent nucleoli (Figure 1C). Three cases demonstrated only cystic degenerative debris with no epithelial cells identified on either LBC or direct smears. In such cases, cell block preparation was performed and revealed diagnostic oncocytic cell clusters, confirming the diagnosis of Warthin tumors (Figure 1D). Oncocytoma/NOH cases cytologically featured cohesive organoid or acinar clusters of oncocytes, wrapped by a fine vascular network, set in a clean or bloody background. Preparation methods revealed distinct architectural differences: LBC samples presented as compact clusters with distinct organoid arrangement resembling a “bunch of grapes” (Figure 2A), whereas direct smears exhibited branching to cohesive clusters supported by a delicate vascular network, mixed scattered small to large clusters in organoid architecture (Figure 2B). The nuclei are round, uniform, and centrally located with small but distinct nucleoli (Figure 2C,D). The cytoplasm is characteristically dense and granular cytoplasm with a low N/C ratio. No cytoplasmic vacuolization or extracellular matrix was present. The two available cell blocks contained tissue fragments composed of oncocytes arranged in an organoid pattern (Figure 2E). These features correlate precisely with the final histology (Figure 2F).

**TABLE 2:** Cytomorphologic features in each case.

Cases	Milan category	Cellularity	Architecture	Cellular features	Background	Histologic diagnosis
1–39	Milan IVa	Low to adequate	Cohesive, flat clusters of oncocytic cells in both LBC and DS	Low N:C ratio, centrally placed nuclei	Cystic degenerative debris and a lymphocyte‐rich background	Warthin tumor
40–43	Milan IVa	Adequate	LBC: Cohesive, clusters with borders, organoid and acinar patterns. “bunch of grapes” pattern	Low N:C ratio, centrally placed nuclei	Clean or bloody	Oncocytoma/NOH
			DS: branching to cohesive clusters supported by a fine fibrovascular network. Retained organoid architecture			
44–45	Milan IVb	Low	LBC: Cohesive clusters with organoid and acinar patterns.	Low N:C ratio, centrally placed nuclei	Clean	Oncocytoma/NOH
		1–2 clusters of oncocytes (6 to 10 cells per cluster)	DS: Acellular			
46	Milan IVb	Low	LBC: Cohesive, flat cluster with smooth borders	Low N:C ratio, centrally placed nuclei	Clean	Spindle cell lipoma
		1 cluster of oncocytes (15 to 20 cells per cluster)	DS: Few mature adipose tissues. No oncocytic cells seen			
47	Milan IVb	Low	LBC: Cohesive, clusters, organoid and acinar patterns.	Low N:C ratio, centrally placed nuclei	Clean	Oncocytic lipoadenoma
		3 clusters of oncocytes (10–15 per cluster)	DS: Acellular			
48–49	Milan IVb	Adequate	LBC: Irregular, flat, loose clusters. Clusters vary in size, some occurring singly. Loss of organoid arrangement	Low N:C ratio, eccentrically placed nuclei. Extremely rare cytoplasmic vacuoles (Present only in one case).	Bloody	OMEC
			DS: irregular, flat clusters, with some resembling a branching pattern. Discohesion is more prominent. Singly occurring cells are numerous between clusters			

Abbreviations: DS, direct smear; LBC, liquid‐based cytology; NOH, nodular oncocytic hyperplasia; OMEC, oncocytic mucoepidermoid carcinoma.

**FIGURE 1:**
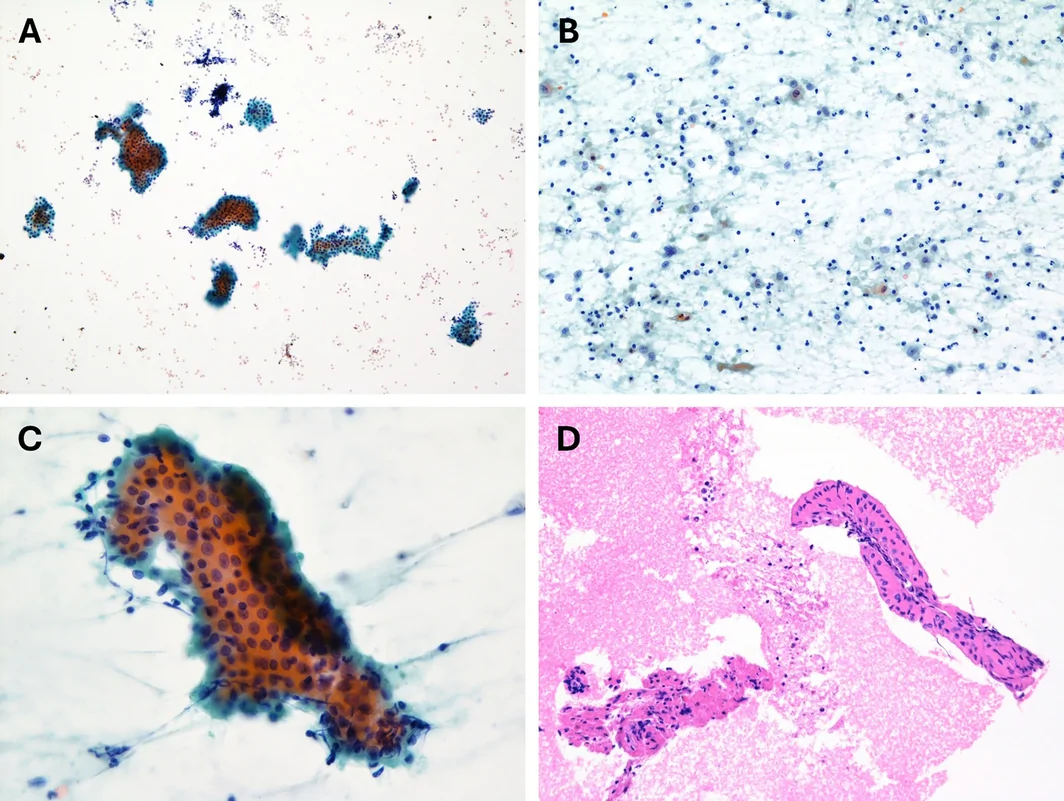
Cytomorphologic features of Warthin tumor. (A) Pap‐stained BD SurePath showing cohesive clusters of flat oncocytic cells in a cystic and lymphocytic background (100×). (B) Conventional smear showing degenerated cystic and lymphocytic background materials (200×). (C) Conventional smear demonstrating cells with round, uniform nuclei with dense, granular cytoplasm (400×). (D) Cell block H&E preparation exhibiting bilayer oncocytic cells absent in LBC and smear preparations (200×). [Color figure can be viewed at wileyonlinelibrary.com]

**FIGURE 2:**
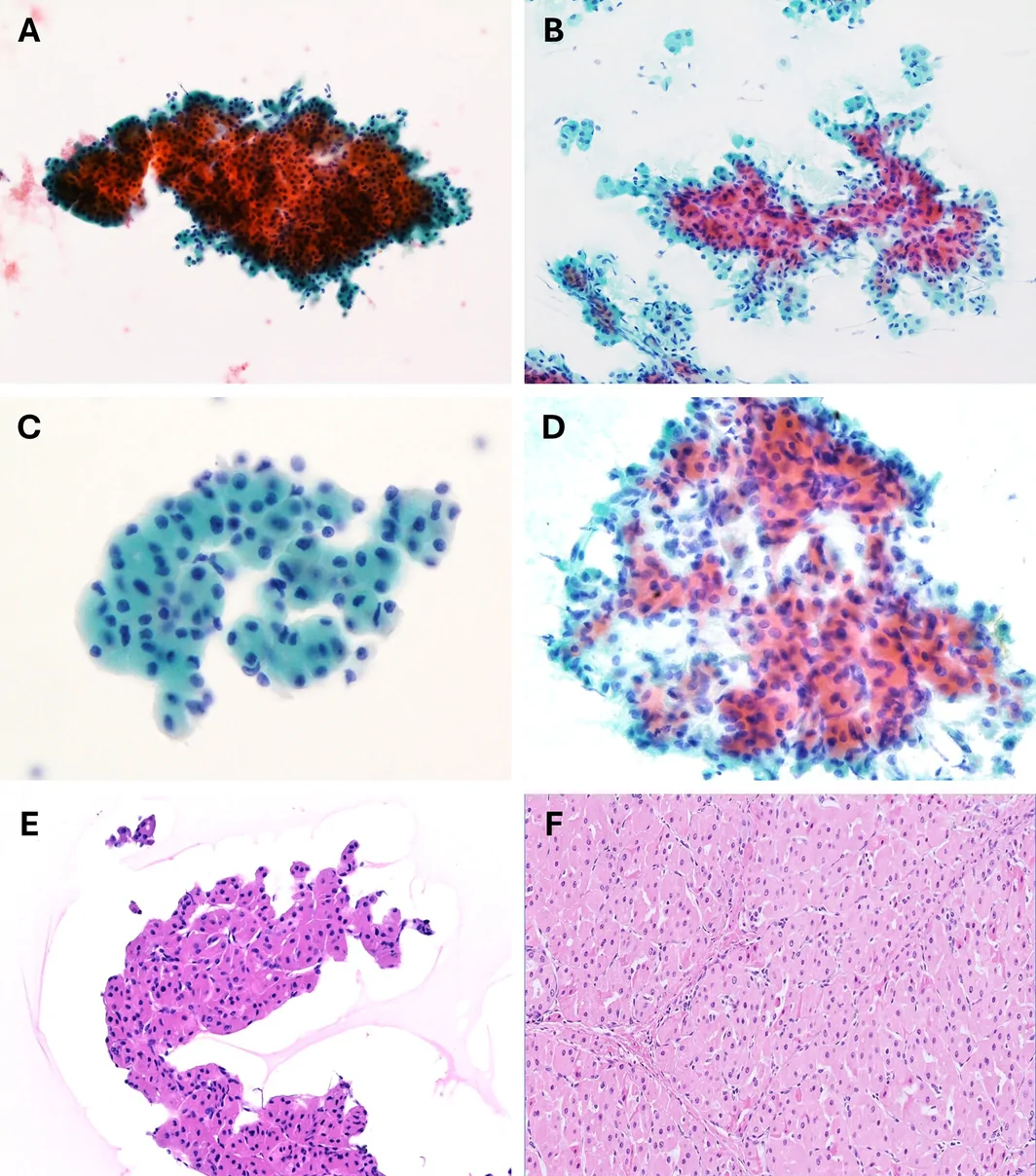
Cytomorphologic features of Oncocytoma/Nodular oncocytic hyperplasia. (A) Pap‐stained SurePath preparation showing compact clusters of organoid arrangement resembling “bunch of grapes” (200×). (B) Pap‐stained direct smear demonstrating mixed branching and tight clusters in organoid arrangement (200×). (C) Pap‐stained SurePath preparation demonstrating cells with round, uniform, and centrally located nuclei with dense, granular cytoplasm (400×). (D) Pap‐stained direct smear. Note the vasculature surrounding each acinar cluster (400×). (E) H&E‐stained cellblock preparation and (F) Tissue section showing oncocytoma (200×). [Color figure can be viewed at wileyonlinelibrary.com]

Within the Milan IVb oncocytic SUMP group, four histologically benign cases demonstrated paucicellularity insufficient for definitive diagnosis. Specifically, two oncocytomas and one oncocytic lipoadenoma yielded only 1–3 small oncocytic clusters; these exhibited an organoid pattern and were identifiable only on LBC preparations, as both direct smears and cell blocks were non‐contributory. In the case of spindle cell lipoma, LBC revealed a single small oncocytic cluster with smooth edges, while the direct smear contained only adipose cell aggregates. Although scant, the cytologic features of the oncocytes in the oncocytoma and oncocytic lipoadenoma cases correlated with the final diagnoses. However, the oncocytic lipoadenoma lacked a discernible lipomatous component on cytology, and the spindle cell lipoma was ultimately discordant, as no oncocytic cells were identified upon final histologic review.

The two OMEC cases were predominantly cellular and presented within a clean background. While both LBC and smear preparations exhibited features overlapping with benign oncocytic lesions, several subtle yet diagnostically important findings were identified. On LBC, tumor cells primarily formed loosely cohesive, irregular, and flat clusters with a notable loss of organoid architecture. Furthermore, both cases demonstrated discohesion, characterized by a tendency for tumor cells to detach from larger clusters, yielding smaller, irregular sheets and scattered, isolated single cells (Figure 3A). Individual cells in both preparations exhibited eccentric nuclei with minimal‐to‐moderate atypia, within a characteristically dense, granular cytoplasm of variable volume. Notably, one OMEC case demonstrated an extremely rare but unequivocal cytoplasmic vacuole in a single cell on the direct smear (Figure 3B). The cell block proved highly diagnostic (Figure 3C), unmasking gland‐like structures and intracytoplasmic vacuoles that were virtually occult on routine cytological preparations. All cytological findings correlated precisely with the final histological diagnosis (Figure 3D).

**FIGURE 3:**
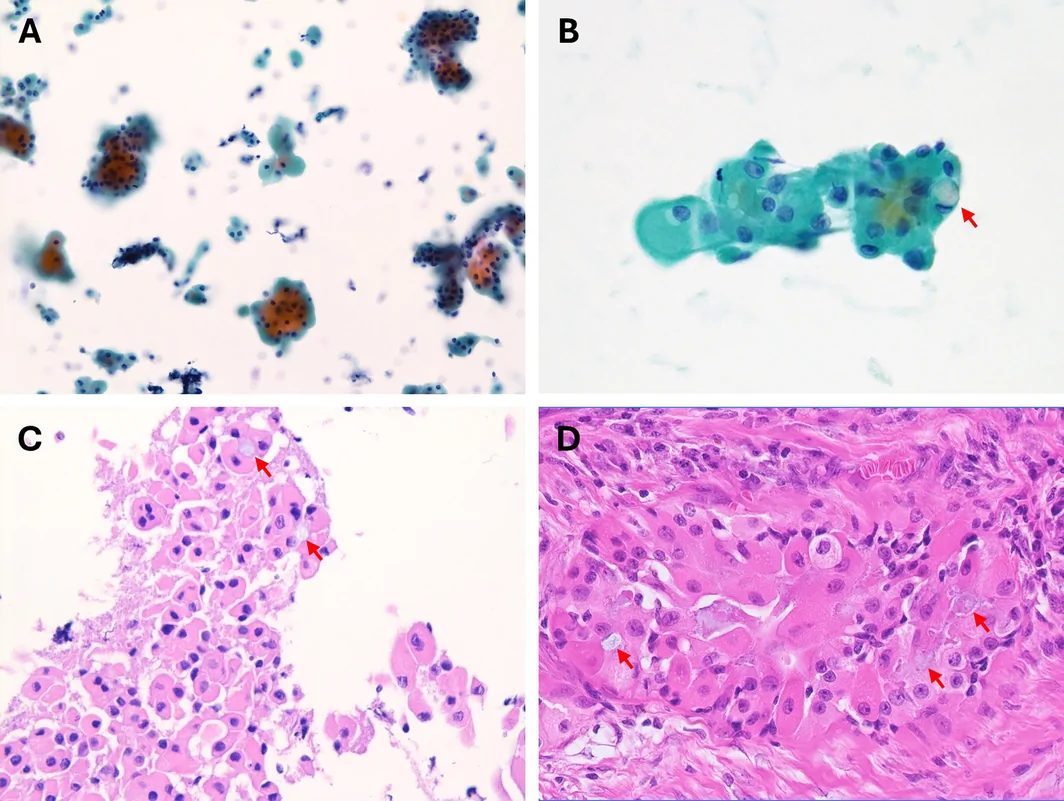
Cytomorphologic features of Oncocytic Mucoepidermoid carcinoma. (A) Pap‐stained SurePath preparation showing loosely cohesive, irregular clusters. Note the scattered single cells (200×). (B) Pap‐stained SurePath preparation showing a cluster with intracytoplasmic mucin (red arrow) (400×), (C) H&E‐ stained cellblock showing oncocytic cells in sheets, some in a gland‐like arrangement with mucin (red arrows) (200×). (D) Tissue sections showing oncocytic mucoepidermoid carcinoma with mucin (red arrows) (400×). [Color figure can be viewed at wileyonlinelibrary.com]

## 4 Discussion

The MSRSGC stratifies the ROM for each diagnostic category and guide clinical management [8]. However, oncocytic lesions remain a significant diagnostic challenge due to the morphologic overlap between benign entities, such as oncocytoma, and malignancies with low‐grade features, specifically the OMEC [12]. In this study, all cases classified as Milan Category IVa (benign oncocytic neoplasm) demonstrated 100% cytohistologic concordance and a 0% ROM. In contrast, cases categorized as Milan Category IVb SUMP exhibited a broader diagnostic spectrum and a higher ROM of 33.3%. However, these findings reflect the experience of a single institution with a relatively small sample size; therefore, the estimated ROM for the SUMP category should be interpreted with caution. Despite this limitation, the observed trends are consistent with the risk stratification objectives of the MSRSGC. Nevertheless, published studies have reported a wider differential diagnosis within the oncocytic SUMP category, with Warthin tumor and conventional mucoepidermoid carcinoma most frequently representing the benign and malignant outcomes, respectively [9, 10, 11]. In contrast, our differentials were restricted to three benign entities (oncocytoma, spindle cell lipoma, and oncocytic lipoadenoma) and one malignant entity (OMEC). The narrower differential in our study is likely attributable to a strict adherence to pure oncocytic morphology, which inherently excludes more heterogeneous lesions and allows for a more focused evaluation of the “oncocytic gray zone.”

Warthin tumor is the second most common benign salivary gland neoplasm after pleomorphic adenoma. It occurs almost exclusively in the parotid gland, although rare cases have been reported in the submandibular gland and cervical lymph nodes. Warthin tumor is strongly associated with chronic cigarette smoking. Histologically, it is characterized by a well‐circumscribed lesion with papillary and cystic structures lined by a bilayer of oncocytic epithelial cells supported by a dense lymphoid stroma. Cytologically, Warthin tumor typically demonstrates flat sheets or single to bilayered clusters of oncocytic cells in a background of cystic degenerative debris and/or abundant lymphocytes [13]. In the present series, these characteristic cytomorphologic features were identified in the majority of cases. However, a subset of cases exhibited absent or only scant diagnostic features, precluding a definitive cytologic diagnosis. In these instances, correlation with the patient's smoking history and evaluation of cell block preparations were instrumental in establishing the diagnosis. Cases showing only background material without an epithelial component, even after cell block preparation, and with compatible clinical findings were classified as Milan Category I (non‐diagnostic). This approach helped maintain a high cytohistologic concordance rate while minimizing the use of indeterminate diagnostic categories. Although uncommon, Warthin tumor may exhibit squamous and mucinous metaplastic changes that overlap cytologically and histologically with variants of mucoepidermoid carcinoma, including OMEC and Warthin‐like mucoepidermoid carcinoma [14, 15]. These metaplastic changes were not encountered in the present series, representing a limitation of the study. In cases with overlapping morphologic features, classification as Milan Category IVb (SUMP) and/or the use of ancillary studies is recommended to avoid underdiagnosis and to account for these diagnostic possibilities.

Oncocytoma of the salivary gland is a rare benign lesion that most commonly arises in the parotid gland, accounting for approximately 80% of cases [12]. Histologically, it is typically well circumscribed and encapsulated, with organoid or nested arrangements of oncocytic cells separated by a delicate vascular network. In contrast, NOH is usually multinodular and unencapsulated. On FNA, however, these entities are practically indistinguishable, as both are characterized by cohesive sheets or clusters of oncocytic cells with well‐defined cytoplasmic borders in a clean or bloody background [8]. In our series, the diagnosis was supported by cytohistologic correlation, with cohesive organoid clusters serving as a reliable diagnostic feature. A notable observation was the preparation‐dependent morphology: direct smears demonstrated a vascular‐rich, branching “bunch of grapes” pattern, whereas liquid‐based cytology preparations tended to produce compact clusters with well‐defined borders. Despite these distinct appearances, both patterns accurately reflected the underlying organoid architecture confirmed by cell block and surgical resection, supporting the observations of Ferreira et al. [16] These cytologic features of oncocytoma were consistent across preparations and may therefore represent more reliable diagnostic features.

Diagnostic challenges were particularly pronounced in Category IVb (SUMP). Diagnostic challenges were particularly pronounced in Category IVb (SUMP). In this category, the distinction between benign and malignant lesions relies almost exclusively on architectural features. Consequently, paucicellular samples are inherently suboptimal for evaluation, as they limit the assessment of tissue architecture and preclude exclusion of artifactual architectural distortion resulting from sampling. In the cases of oncocytoma and oncocytic lipoadenoma, the presence of only sparse clusters, identified exclusively on LBC, and the lack of architectural context precluded a definitive benign diagnosis. In such scenarios, cellularity is insufficient for definitive characterization, yet clinical or radiologic evidence strongly suggests a neoplasm; classifying these cases as SUMP is recommended [8]. This approach ensures diagnostic safety by acknowledging the likely neoplastic nature of the lesion while accounting for the inherent limitations of a paucicellular sample. Distinguishing oncocytic lipoadenoma from an oncocytoma is difficult, as both contain oncocytic cells arranged in organoid clusters. Even when lipomatous components are present, they may be misinterpreted as normal parotid parenchyma or surrounding adipose tissue. In such instances, clinical correlation with imaging becomes vital; a well‐defined, heterogeneous lesion on CT or MRI may raise suspicion for oncocytic lipoadenoma, whereas oncocytoma typically exhibits more homogeneous imaging characteristics [12, 17]. This category also harbors a risk of cyto‐histological discordance. In the case of spindle cell lipoma, oncocytic clusters identified on cytology were absent on final histology. This discrepancy can likely be explained by the sampling of focal oncocytic changes or reactive oncocytosis adjacent to the lesion during aspiration. However, because no surrounding tissue was included in the surgical specimen, this hypothesis remains difficult to prove.

The most critical distinction in this series lies in separating benign oncocytoma from OMEC. These are summarized in Table 3. While OMEC can mimic benign architecture, it often displays subtle diagnostic “red flags,” including cell dissociation, eccentric nuclei, and variations in cytoplasmic volume, which may impart a pleomorphic appearance even in the absence of high‐grade nuclear features. Our findings align with the existing literature: while some authors do not explicitly define these features as diagnostic criteria, they are consistently reported in published histopathologic and cytologic imaging [3, 12, 18, 19, 20]. Interestingly, these features are not exclusive to OMEC. Other oncocytic/oncocytoid tumors, such as oncocytic epithelial‐myoepithelial carcinoma, oncocytic salivary duct carcinoma, and acinic cell carcinoma, may also demonstrate cell dissociation, in addition to entity‐specific features [8, 20, 21, 22]. This suggests that, in the context of low‐grade oncocytic lesions, these features may serve as more sensitive markers of malignancy than traditional criteria such as atypia, mitoses, or necrosis. Another potential pitfall is the presence of intracytoplasmic vacuoles and a mucinous background. These are remarkably rare and difficult to identify on routine smears, with the latter being virtually absent. Since these features can be seen in both benign (Warthin tumor with mucinous metaplasia) and malignant lesions (OMEC), they are not reliable in differentiating these lesions [12].

**TABLE 3:** Cytomorphologic comparison between onocytoma/NOH and OMEC.

Cytomorphologic features	Oncocytoma/NOH	OMEC
Architecture	Cohesive to branching, organoid and acinar patterns resembling “bunch of grapes.”	Loosely cohesive clusters. May form irregular flat sheets or scattered single cells.
Cytology	Uniform, centrally‐placed nuclei, abundant, dense, granular cytoplasm, low N/C ratio	Uniform, occasionally eccentric nuclei, with mild to moderate atypia, variation in cytoplasmic volume, characteristically dense and granular, low N/C ratio
Background matrix/material	Clean or bloody	Clean or bloody
Cell block	Cohesive organoid clusters.	Loosely cohesive clusters, rare intracytoplasmic mucin.

Abbreviations: NOH, nodular oncocytic hyperplasia; OMEC, oncocytic mucoepidermoid carcinoma.

Cell block emerged as a pivotal diagnostic tool; in one OMEC case, it alone revealed the gland‐like structures and cytoplasmic vacuoles. This underscores the necessity of integrating architectural data to overcome the inherent limitations of FNA cytology, effectively bridging the gap between cytologic suspicion and histologic confirmation. The utility of the cell block extends beyond architectural assessment by providing a platform for immunohistochemical and molecular profiling. While benign oncocytomas typically exhibit only focal or peripheral p63 staining, which highlights myoepithelial cells, OMEC often shows diffuse, strong p63 or p40 expression on tumor cells [23]. Identification of *MAML2* rearrangements identified via break‐apart FISH confirmed the diagnoses of OMEC in this series and remains the gold standard [24]. This underscores a critical diagnostic workflow: recognizing subtle architectural dissociation and cytoplasmic pleomorphism as triggers for molecular testing. Radiologic correlation also provides valuable complementary information: whereas oncocytomas typically present as well‐circumscribed, predominantly homogeneous masses on CT or MRI, low‐grade OMEC may demonstrate infiltrative or irregular margins, consistent with the findings of the series [25, 26, 27].

In conclusion, distinguishing Warthin tumor and oncocytoma from low‐grade OMEC on salivary gland FNA remains a significant diagnostic challenge because of their overlapping cytomorphologic features. Our findings suggest architectural and cytologic features, including flat clusters, loss of organoid pattern, eccentric nuclei, and cellular dissociation, should serve as important diagnostic clues warranting classification as SUMP (Category IVb) rather than a benign neoplasm. The addition of cell block architectural assessment and molecular confirmation by FISH further enhances diagnostic accuracy and clinical management.

## Author Contributions

**Lois Araneta:** data curation, formal analysis, investigation, and writing – original draft. **Ying‐Ju Kuo:** data curation, investigation. **Jen‐Fan Hang:** conceptualization, data curation, investigation, project administration, supervision, and writing – review and editing.

## Funding

The authors have nothing to report.

## Conflicts of Interest

The authors declare no conflicts of interest.

## Data Availability

The data that support the findings of this study are available from the corresponding author upon reasonable request.
